# Cosmogenic soil production rate calculator

**DOI:** 10.1016/j.mex.2019.11.026

**Published:** 2019-11-28

**Authors:** Ángel Rodés, Daniel L. Evans

**Affiliations:** a Scottish Universities Environmental Research Centre, East Kilbride, UK; b Lancaster Environment Centre, Lancaster University, Lancaster, Lancashire, UK

**Keywords:** Cosmogenic soil production rate calculator (CoSOILcal), Cosmogenic nuclides, ^10^Be, ^26^Al, ^21^Ne, ^3^He, ^36^Cl, ^14^C, MATLAB, Octave, Soil production, Erosion rate

## Abstract

To understand the rates at which soils form from bedrock, it is important to know the rates at which the bedrock surface lowers (the apparent erosion rate, which is assumed to be constant). Previous models that calculate apparent erosion rates using measured concentrations of cosmogenic radionuclides rely on the assumption that the bulk density of the soil which forms as a product of bedrock erosion either equals that of the bedrock itself or is constant with depth down the soil profile. This assumption fails to recognise that soils have significantly lower densities that might not be constant with depth. The model presented here allows for the calculation of isotopically-derived soil production rates, considering the bulk density profile of the soil overlying the bedrock surface. This calculator, which can be run both in MATLAB® and GNU Octave©, represents a novel and significant contribution to the derivation of soil production rates.

Specification Table

**Table:** 

Subject Area:	Earth and Planetary Sciences
More specific subject area:	geochemistry; geochronology; cosmogenic nuclides; ^10^Be; ^26^Al; ^21^Ne; ^3^He; ^36^Cl; ^14^C
Method name:	Cosmogenic soil production rate calculator (CoSOILcal)
Name and reference of original method:	Evans, D. L., Quinton, J. N., Tye, A. M., Rodés, Á., Davies, J. A. C., Mudd, S. M., and Quine, T. A (2019) Arable soil formation and erosion: a hillslope-based cosmogenic-nuclide study in the United Kingdom, *SOIL, 5, 253-263.*https://doi.org/10.5194/soil-5-253-2019
Resource availability:	set of scripts submitted with this paper

## 1 Method details

Herewith we present a set of MATLAB® / GNU Octave© scripts and their mathematical description. These are designed to calculate the surface erosion rates using one or more measured cosmogenic concentrations at or below the surface when the bulk density profile is known. An example of this model's application is described in [4].

### 1.1 Input data

Site data has to be inputted in individual comma separated files (.csv) for each site. An example of input file is attached (see “input_data.csv”). The input file contains the following headers (first line) that we recommend are not changed:

1 **Depth**: List of depths where density was measured or where samples were collected for cosmogenic radionuclide analysis. In cm.
2 **Density**: Measured densities in g/cm^3^
3 **Concentration**: Measured concentrations of *in-situ* cosmogenic ^10^Be, ^26^Al, ^21^Ne, ^3^He, ^36^Cl or ^14^C in atoms/g.
4 **Concentration Uncertainty**: Uncertainty of the cosmogenic isotope concentration in atoms/g.
5 **Isotope mass**: Atomic mass of the measured isotope: 10, 26, 21, 3, 36 or 14.
6 **Lat. (Degrees)**: Latitude of the sampled site, to be inputted only in the second line of the csv file.
7 **Lon. (degrees)**: Longitude of the sampled site, to be inputted only in the second line of the csv file.
8 **Elv. (m)**: Elevation of the sampled site, to be inputted only in the second line of the csv file.
9 **Shielding**: Shielding factor at the surface of the sampled site, to be inputted only in the second line of the csv file.
10 **Landscape age (a)**: Known age of the landscape in years. Input a large number (e.g. the age of the Earth: 4.54E+09) to consider steady state conditions. Only in the second line of the csv file.

Please leave the cells without data empty (i.e. do not put zeros) and place the desired csv files in the same folder as the scripts (by default in the CoSOILcal folder).

### 1.2 Model fit

**Fig. 1:**
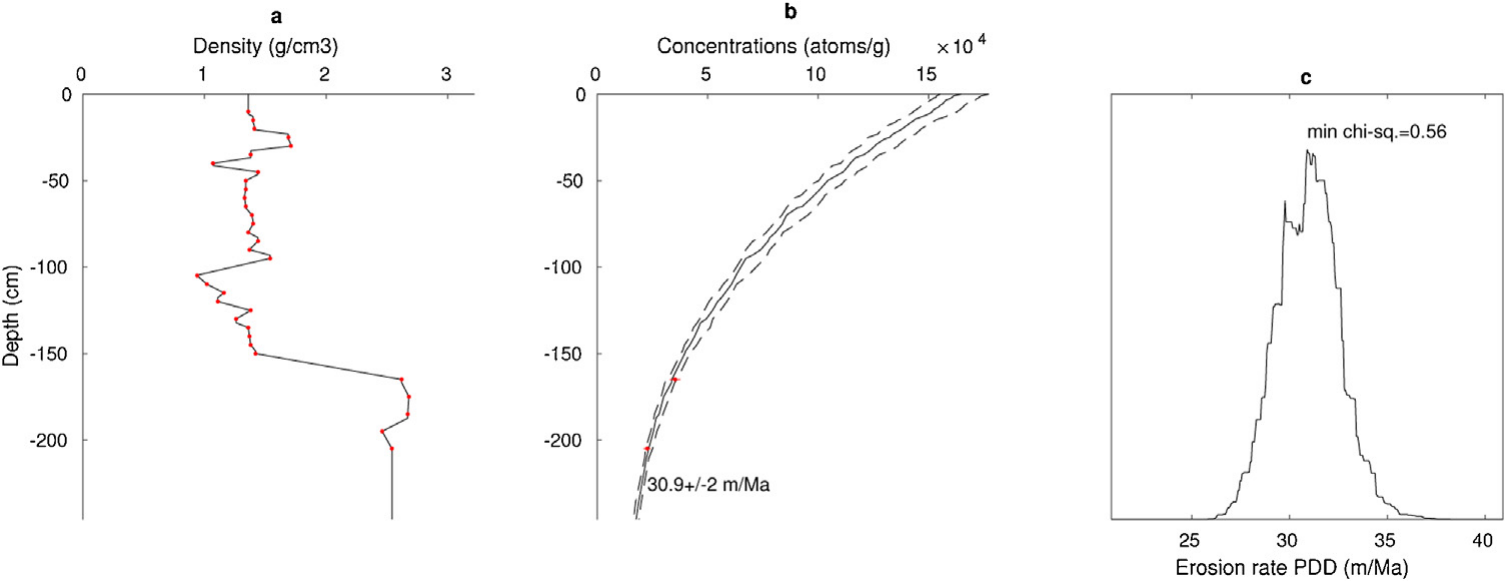
**Graphical abstract.** The graphical output of CoSOILcal includes (a) the considered density profile (measured densities as red dots), (b) the measured cosmogenic concentrations (red) and the depth profile of modelled concentrations in black (dashed lines reflect the results within one-sigma), and (c) the probability density distribution (PDD) of the modelled erosion rates.

To model the apparent erosion rates, associated uncertainty and the graphical output shown in Fig. 1, just run the script start.m and select the desired csv file(s) in the pop-up dialog.

## 2 Under the hood

The mathematical details of the calculations made in each script are described here:

### 2.1 start.m

This script generates the dialog that allows selecting the input file(s) and calls soil_solver.m for each dataset.

### 2.2 soil_solver.m

This is the main function. Depth (*z*), density (*ρ*) and effective mass depth (*x*) arrays are generated and are logarithmically distributed between 1 cm and 100 m, including all the depths that contain input data (density measurements or cosmogenic concentrations). Densities outside the measured range are extrapolated using the shallowest and deepest measurements. The rest of the densities is interpolated from the nearesr neighbours, as shown in Fig. 1a. The effective mass depth (*x*) is calculated as

(1) $$x(z)=\sum_{z_{i}=0}^{z}\Delta z_{i}\cdot \rho$$

The surface production rates for cosmogenic isotopes are calculated using the Production_rate.m function, described below. Modelled cosmogenic concentrations and deviation from the data are calculated with the model.m and desvmodel.m functions, also described below.

Erosion rates (*ε*) are fitted iteratively by the interpolation of a variable erosion rate array, starting with erosion rates logarithmically distributed between 1 cm/a and 100 m/Ma. Best fit and one sigma upper and lower bounds are plotted in Fig. 1b.

Chi-square values (*χ*^2^) are calculated as the sum of the squared deviations. Models with chi-squared values smaller than the minimum chi-square value plus the number of samples are considered to fit the data within one-sigma confidence level. Relative probabilities associated to the chi-squared values are calculated as $e^{\left(-\chi^{2}/2\right)}$. These probabilities are plotted in Fig. 1c.

### 2.3 Production_rate.m

This function calculates these surface production rates, the apparent attenuation lengths of fast and stopping muons under the surface, and the corresponding pressure for a given latitude, longitude and elevation. It uses the following code from the CRONUS calculators v.2.3 [2]: NCEPat_2.m and NCEP2.mat to calculate pressure, antatm.m to calculate pressure in Antarctica (if latitude <−55), al_be_consts_v23.mat for constants, stone2000.m and PMag_Mar07.mat for spallation production rates, and P_mu_total.m for muon production rates at the surface.

The inputs of this function are *site_lat*, *site_lon*, *site_elv*, *shielding* and *nuclide*, as defined in section 1.1.

Calculated ^10^Be production rates in quartz are scaled for other isotopes based on published ratios:

• ^26^Al/^10^Be production rate ratios in quartz are taken from [2]
• ^21^Ne/^10^Be production rate ratios in quartz are taken from [1]
• ^3^He/^10^Be production rate ratios in pyroxenes and quartz are taken from [3]
• ^36^Cl/^10^Be production rate ratios in calcite and quartz are taken from [5]
• ^14^C/^10^Be production rate ratios in quartz are taken from [5]

**Fig. 2:**
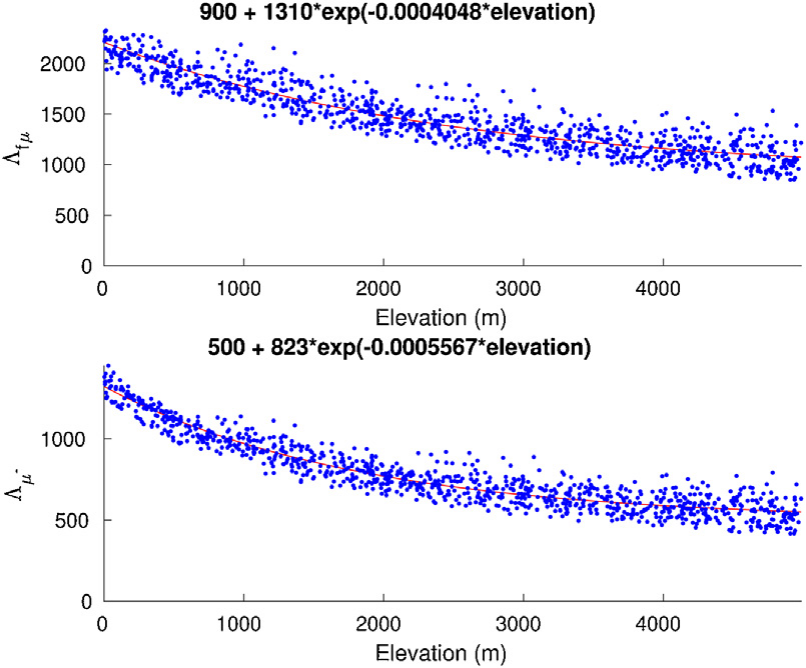
Apparent muon attenuation lengths. A thousand muon-production depth profiles between 2 and 10 m around the globe were generated using P_mu_total.m. 100 muon production rates were calculated for each profile. Apparent muon attenuation lengths (blue dots) were calculated by fitting production rates to exponentials. The calculated relations between apparent attenuation lengths and elevation (red lines) fit the synthetic data within a 7%. This 7% uncertainty is mostly due to the variability in the depth of the randomly generated profiles.

Apparent muon attenuation lengths can be calculated by fitting muon production rates at different depths (from P_mu_total.m) to simple exponentials. A thousand depth profiles were randomly generated for maximum depths between 2 and 10 m around the globe. Resulting values of the apparent attenuation lengths were they fitted to altitude exponentials as shown in Fig. 2. The following approximations fit the apparent attenuation lengths within a standard deviation of 7%:

(2) $$\Lambda_{f\mu }=900+1310*e^{-0.0004048\cdot h}$$

(3) $$\Lambda_{\mu^{-}}=500+823*e^{-0.0005567\cdot h}$$

where Λ_*fμ*_ and $\Lambda_{\mu^{-}}$ are the attenuation lengths of fast and stopping muons in g/cm^2^ respectively and *h* is the elevation of the site.

The outputs of this function are: Production rates, attenuation lengths and atmospheric pressure.

### 2.4 model.m

This function calculates the cosmogenic isotope concentration at several depths (*zs*) considering the surface production rate data and decay rate (*P*, Λ, *λ*), a variable-density profile (*z*, *x*) for a landscape age and several erosion rates (ε). The time (*t*) is discretized in an array of 100 values logarithmically distributed between 100 years and the landform age. For each *t*, *zs* and *ε* combination, a corresponding effective depth *x* is calculated by interpolation of *zs* + *ε* · *t* in the variable-density profile. Then the accumulated cosmogenic concentration is calculated following:

(4) $$C_{i}=\sum_{\mathrm{Sp}.,f\mu ,\mu^{-}}\frac{P}{\lambda +\frac{\varepsilon \cdot \rho }{\Lambda }}\cdot e^{-x/\lambda }\cdot \left(1-e^{-\Delta t\cdot (\lambda +\frac{\varepsilon \cdot \rho }{\Lambda })}\right)$$

where *C*_*i*_ is the concentration accumulated during Δ*t* time step at the mass depth *x*, *epsilon* is the erosion rate of the surface and *ρ* is the average density at the depth *ε* · *t* for the time frame from *t* − Δ*t* to *t*.

Finally, all the calculated concentrations for the 100 time steps are summed as:

(5) $$C=\sum_{t=0}^{T}C_{i}\cdot e^{-\lambda \cdot t}$$

where *T* is the landform age.

### 2.5 desvmodel.m

This function calculates the deviation of a model respect a set of cosmogenic isotope concentrations as:

(6) $$s=\sum \frac{C-M}{\sigma_{M}}$$

where *C* is the model concentration, *M* is the measured concentration and *σ*_*M*_ is the uncertainty of measured concentrations. The inputs are the same as in model.m plus a set of sample depths, measured concentrations and their uncertainties. This function accepts erosion rates as an array of values.

## Supplementary material

All scripts discussed in section 2 are included in the CoSOILcal_v1_0.zip file.
